# Person-centred care in chronic kidney disease: a cross-sectional study of patients’ desires for self-management support

**DOI:** 10.1186/s12882-016-0416-2

**Published:** 2017-01-13

**Authors:** Kathryn Havas, Clint Douglas, Ann Bonner

**Affiliations:** 1 School of Nursing, Queensland University of Technology, Victoria Park Rd, 4059 Kelvin Grove, Brisbane, QLD Australia; 2 Chronic Kidney Disease Centre for Research Excellence, University of Queensland, Brisbane, Australia; 3 Visiting Research Fellow, Kidney Health Service, Metro North Hospital and Health Service, Brisbane, Australia

**Keywords:** Chronic kidney disease, Self-management, Patient education, Patient-centred care, Patient preferences

## Abstract

**Background:**

People with chronic kidney disease (CKD) must self-manage their illness to assist with slowing disease-progression, but this is a complex task requiring support from healthcare professionals. Despite the established importance of person-centred care, people with CKD are rarely consulted regarding their desires for self-management support (SMS).

**Methods:**

A cross-sectional survey was conducted face-to-face in a Queensland primary care clinic and distributed Australia-wide via an online interface promoted by Kidney Health Australia during 2015. Participants were ≥18 years old and had a self-reported doctor’s diagnosis of CKD (any stage; *N* = 97). The survey was based upon existent literature which identified 10 areas that those with CKD believe require additional support. Descriptive data were generated and Mann-Whitney U tests were performed to compare the desires of different groups of participants.

**Results:**

Of the 97 participants, 36 completed a hardcopy survey in clinic, and 61 completed the online version. Just over half (60.8%) were female, age ranged from 16–89 (*M* = 56.44), and time since diagnosis ranged from just diagnosed to 60 years (*Mdn* = 8.08 years). Strong interest in receiving additional support across all 10 areas was reported (*Mdns* = 8.00–10.00), with “keeping a positive attitude and taking care of mental and physical health” receiving the highest rating. Those who were: younger (*p* < .001); more highly educated (*p* < .001); working (*p* < .001); diagnosed longer ago (*p* = .015); and women (*p* = .050) expressed stronger overall desire for additional support.

**Conclusions:**

In addition to information about CKD and medications, everyday strategies ought to be prioritised in patient education. Varying levels of engagement and eagerness to learn more about self-management highlight the need for a person-centred approach to SMS.

**Electronic supplementary material:**

The online version of this article (doi:10.1186/s12882-016-0416-2) contains supplementary material, which is available to authorized users.

## Background

People with chronic kidney disease (CKD) must self-manage their illness to a large extent to slow disease-progression by effectively managing medications and making lifestyle modifications (e.g., diet, fluid, exercise, smoking, alcohol). Engaging in this level of self-management is arduous and requires support, however, not all individuals desire the same kind or level of assistance [1]. Despite this, people with CKD are rarely consulted regarding the support that they desire in order to effectively manage their condition [2]. This is incongruent with person-centred care, which entails involving the individual as an active participant in their healthcare (i.e., patient engagement) and tailoring treatment to individual wants and needs [3–5]. This model of care can help to achieve optimal outcomes for patients [6, 7] and is also recognised as an important indicator of quality of service provision [8–13]. The specialty of nephrology has been late to recognise the importance of person-centred care and patient engagement [14]; however the potential of person-centred care to complement evidence-based practice in providing healthcare for people with CKD is beginning to receive greater attention [15–17].

CKD self-management programs/interventions can lead to positive outcomes including improvements in: symptoms/problems [18]; disease-specific knowledge [19]; self-care knowledge, ability, and behaviour [20, 21]; health-related quality of life [22]; interdialytic weight gain [23], blood pressure, and laboratory measures [24] as proxies for adherence to treatment; psychological problems [25]; health service utilisation [26]; time to initiation of renal replacement therapy (RRT [27]); and survival after beginning RRT [28]. Participants of self-management programs also report finding them helpful [29], and those who have not yet participated in such a program express interest and a willingness to fit appointments into their (often already appointment-heavy) lives [30]. However, in designing an intervention for people with CKD, it is important that the needs and preferences of the target population are considered, rather than applying a “one-size-fits-all” approach based upon clinician perspectives of what people with CKD *should* receive.

Previous research in CKD has found that patients report a lack of practical, individualised self-management support (SMS) from their healthcare providers (HCPs [16, 17]). Patients also express frustration at lack of HCP engagement with their SMS needs and lack of explanation of why lifestyle modifications and/or treatments are important [31–33].

A recently published review of 12 studies which sought the perspectives of people with CKD [2] identified 10 broad areas of self-management (see Table 1). The aim of this exploratory study was to directly assess the extent to which people with CKD wish to receive support with these 10 areas, as well as to identify further areas of self-management requiring support. Furthermore, the study aimed to investigate the preferences of patients for how they would like to receive this support (format, educator, time, and location). In keeping with the principles of person-centred care, this research also aimed to explore how the support needs of people with CKD vary based upon background characteristics.

**Table 1:** Areas of CKD self-management previously identified as requiring additional support [2]

Aspect of Self-Management Support	Description
1. Disease-specific knowledge	Information about what kidneys do, how they work, what happens in CKD, treatment options, ways to delay dialysis.
2. Managing medications	Understanding why medications are prescribed, possible side effects, what might happen if not taken, how to take them as prescribed.
3. Engaging and sustaining social support	Engaging with friends and family to get CKD support, becoming involved with community groups including support groups.
4. Maintaining social and occupational roles	Continuing to work, sustaining hobbies, maintaining relationships and home roles.
5. Modifying lifestyle	Adhering to fluid and dietary guidelines, engaging in appropriate physical activity.
6. Developing and sustaining a positive attitude and caring for mental and physical wellbeing	Avoiding anxiety and depression, staying positive, staying generally physically healthy.
7. Building and sustaining effective relationships with healthcare providers	Developing effective working relationships with doctors, nurses, clinic staff, allied health professionals, and any other members of and individual’s healthcare team.
8. Establishing routine and planning ahead	Getting into good self-management habits, putting effective strategies such as reminder systems in place.
9. Actively participating in healthcare	Learning to change self-management behaviour based upon results, working collaboratively with HCPs regarding CKD and its treatment.
10. Recognising and effectively responding to symptoms	Noticing signs and symptoms of CKD and knowing what to do when they occur, learning to avoid worsening CKD symptoms.

## Methods

A cross-sectional, self-administered survey was conducted between March and November 2015. Participants were recruited from a primary care clinic in Queensland, Australia, and via social media and newsletter advertisements through an Australian not-for-profit CKD support organisation. Those recruited in clinic completed the survey using pen and paper; those who responded to advertisements completed an identical online version. Collecting data in these two different modes allowed us to include a diverse sample in terms of age, level of education, income, and CKD stage/time since diagnosis. Furthermore, collecting data in a face-to-face, one-on-one session for over one-third of participants (while time and resource intensive) allowed for the inclusion of those who are quite often missed in survey research [34]. For example, one participant was illiterate, and some could speak and understand English but were unable to read it. Collecting data in this manner meant that questions could be read aloud to these participants, and their answers could be included. Ethical approval was obtained via the Queensland University of Technology Human Research Ethics Committee (EC00171). All participants received detailed study information, and voluntary completion and submission of the survey constituted informed consent. All participants reported a doctor’s diagnosis of CKD (any stage), were over 18 years of age, and understood English.

The survey consisted of 25 items investigating demographic details and participants’ preferences for receiving SMS, including what they would like to learn and how and from whom they would like to receive support (see Table 2 for a summary of questions). Participants rated the extent to which they would like more support with 10 areas of self-management based on the literature [2] from 0 (not at all) to 10 (very much). Questions regarding format and delivery preferences were either multiple choice or “select as many as apply”. In addition, participants were given room to write any additional suggestions they might have (instrument in Additional file 1).

**Table 2:** Summary of survey questions

Question Summary	
Background information:	How would you like to receive self-management support?
• Gender	• When in the week could you attend sessions?
• Age	• Where could you attend sessions?
• Years of education	• Would you prefer group/individual sessions?
• Highest educational qualification	
• Employment status	• Would you like to bring a friend/family member?
• Occupation (if employed)	
• Annual household income	• Who would you like to receive support from?
• Time since diagnosis	
To what extent would you like to learn more about (rate from 0 = “not at all” to 10 = “very much”):	
• Each of the 10 areas of self-management identified in Table 1	

### Data analysis

Quantitative data were analysed using SPSS version 22. Continuous data are presented as median (interquartile range; IQR). As data failed assumptions of t-tests, non-parametric Mann-Whitney U tests were used. Due to comparison groups having extremely different distribution shapes, results of comparisons are summarised in terms of mean rank. Mean ranks are generated by ranking all scores on the dependent variable across both groups from smallest to largest. The ranks for each group are then averaged, and these averages are compared between groups to assess for statistically significant (*p* < .05) difference.

To compare groups of participants, meaningful cut-offs were employed. For age, participants were categorised as < 60 years vs ≥ 60 years, as those aged 60+ are commonly considered seniors [35]. For education, participants were split into those who had received a grade 10 (or equivalent) education or less vs those who had completed further study, as grade 10 is the point at which Australians can choose to leave school [36]. Income was split into < $39,999 vs ≥ $40,000, so that the first category would capture those on minimum wage and those receiving government support, while the second would capture those on higher salaries. Two analyses were run on time since diagnosis: ≤ 3 years vs > 3 years and < 10 years vs ≥ 10 years. The former analysis was conducted as people with CKD may be more activated to learn more when first diagnosed [37]. The latter was conducted as previous research has revealed that those who have been living with the disease for a long time and are in later stages regret a lack of understanding of the importance of self-management behaviour in the earlier stages of the disease [38]. As such it was hypothesised that these people may have a greater appreciation for the merits of additional support.

A very small amount of simple, textual qualitative data were collected in the form of asking participants if they had any additional suggestions regarding areas that would be important to address during a CKD self-management program and/or how such a program should be run. A two-step process was used with this data. First, suggestions which had already been captured by existing scales were excluded. Remaining data were coded into categories [39], and then deductive content analysis [40] was used to ascertain how many participants had mentioned each identified category.

## Results

### Sample characteristics

A total of 97 participants completed the survey; 36 completed a hard copy in the clinic, while 61 completed the online version. Of these participants, 59 (60.8%) were female, and age ranged from 16 to 89 years old (*M* = 56.44). The survey captured experiences of people across the CKD trajectory, with time since diagnosis ranging from just diagnosed to 60 years (*Mdn* = 8.08 years). Almost half (41; 42.3%) of participants had a grade 10 level of education or below, while the remaining 57.7% (56) were more highly educated. Just over half (51; 52.6%) of participants were working, and the other 46 (47.4%) were unemployed or retired (see Table 3).

**Table 3:** Sample Characteristics

Variable		Frequency (%)
Gender		
Male		38 (39.2)
Female		59 (60.8)
Age Range: 16.00–89.00		
*M =* 56.44	SD = 18.00	
≥ 16 < 25		5 (5.2)
≥ 25 < 40		15 (15.5)
≥ 40 < 60		28 (28.9)
≥ 60 < 80		42 (43.3)
≥ 80		7 (7.2)
Years of Education Range: 6.00–23.00		
*M =* 13.39	SD = 3.81	
Highest Educational Qualification Attained		
No Formal Education		1 (1.0)
Less than Grade 10 Equivalent		14 (14.4)
Grade 10 or Equivalent		26 (26.8)
Grade 12 or Equivalent		4 (4.1)
TAFE Qualification/Certificate/Diploma		29 (29.9)
Undergraduate Degree (Bachelors)		17 (17.5)
Masters Degree		5 (5.2)
Doctoral Degree (Including PhD)		1 (1.0)
Time Since Diagnosis (Self-Reported) Range (months): 0.00–720.00		
*Mdn* = 97.00	IQR = 36.00–246.50	
≤ 12 months		11 (11.3)
> 1 ≤ 3 years		14 (14.4)
> 3 ≤ 5 years		15 (15.5)
> 5 < 10 years		10 (10.3)
≥ 10 years		47 (48.5)
Income		
< $20,000		9 (9.3)
$20,000–$39,999		39 (40.2)
$40,000–$59,999		8 (8.2)
$60,000–$79,999		7 (7.2)
$80,000–$99,999		2 (2.1)
$100,000–$119,999		8 (8.2)
$120,000+		15 (15.5)
Don’t Know/Would Rather not say		9 (9.3)
Employment Status		
Unemployed		7 (7.2)
Casual		3 (3.1)
Part Time		21 (21.6)
Full Time		22 (22.7)
Retired		39 (40.2)
Other (Employed)		5 (5.2)

### Overall preferences for self-management support

**Fig. 1:**
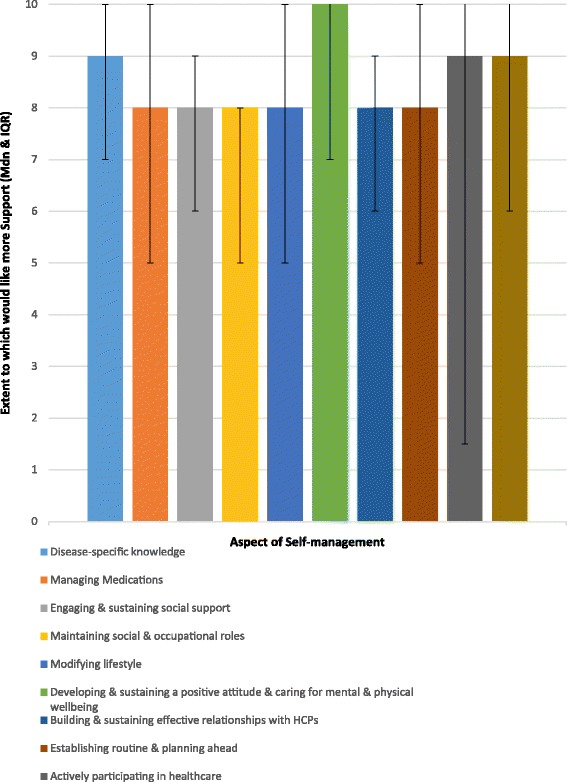
Median ratings of extent to which participants desire additional support with each of the 10 pre-identified areas of self-management. Whisker bars represent interquartile range (IQR)

Overall, participants reported desiring more support with all 10 aspects of self-management. Median ratings for all areas ranged from eight to 10, with the highest median rating (10) given to “keeping a positive attitude and taking care of mental and physical health”. “Actively participating in healthcare”, “CKD-specific knowledge”, and “noticing and treating signs and symptoms” were given median ratings of nine, while the remaining six factes received median ratings of eight (see Fig. 1).

Seven participants identified additional aspects of self-management as requiring extra support. The areas identified (each area identified by one participant) were: libido and sexual health; fertility; learning to track health; managing CKD when living away from support; ways to access new technology; options for respite; managing travel and vacations with CKD; managing off days; and identifying when an appointment with the doctor is indicated. Some face-to-face participants conversationally expressed hopelessness, believing that there is nothing that they can do to help improve their CKD outcomes.

### Differences between participant groups

**Fig. 2:**
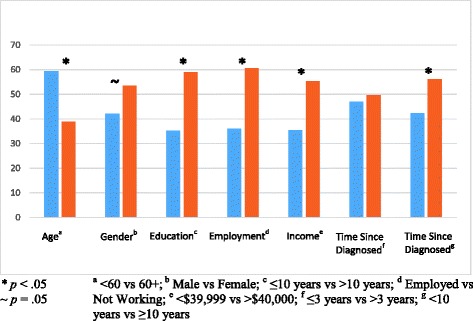
Mean ranks of different groups of participants’ overall desire for more self-management support

We found some groups rated the importance of all (or most) aspects of self-management more highly than others. Because of this, a composite variable “overall desire for additional SMS” was created, by taking the mean of participants’ ratings across the 10 scales. Different subgroups of participants displayed statistically significantly different levels of overall interest in receiving SMS. Younger (vs older) participants (mean ranks = 59.38 and 38.84, respectively, *U* = 678.00, *p* < .001, *r* = .33); those with a higher than grade 10 level of education (vs those who had completed grade 10 or lower; mean ranks = 59.02 and 35.32, respectively, *U* = 1,709.00, *p* < .001, *r* = .42); those who were currently working (vs unemployed or retired; mean ranks = 60.64 and 36.10, respectively, *U* = 1,766.50, *p* < .001, *r* = .44); those earning a higher income (vs those earning a lower income or pension only; mean ranks = 55.34 and 35.47, respectively, *U* = 1,393.00, *p* < .001, *r* = .37); and those who were diagnosed with CKD > 10 years ago (vs those diagnosed within the past 10 years; mean ranks = 56.15 and 42.28, respectively, *U* = 1,511.00, *p* = .015, *r* = .25) expressed higher overall desire for additional SMS. There was a trend approaching statistical significance such that women expressed a stronger desire for SMS than men (mean ranks = 53.48 and 42.04, respectively, *U* = 1,385.50, *p* = .050, *r* = .20; see Fig. 2).

### Preferences regarding support delivery

**Fig. 3:**
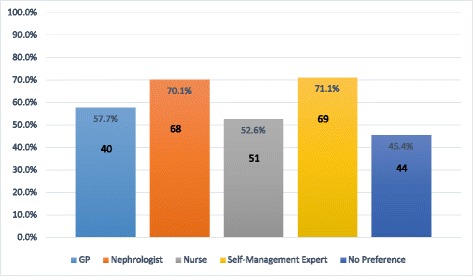
Percentage of participants open to receiving self-management support from different kinds of professionals

When asked about the timing of sessions, most participants (70; 72.2%) expressed that they would be willing to attend SMS sessions during work hours (9–5, Monday-Friday) and about one third (34; 35.1%) would attend sessions after hours (weeknight evenings or weekends). Further analysis revealed that 100% of those who were not working (46) would be willing to attend sessions during work hours, while only 47.1% (24) of those who were working would be willing to do so. Those who were working expressed a preference for weekday evening or weekend sessions, with 58.8% (30) stating they would attend on weekday evenings and 54.9% (28) reporting that they would be willing to attend weekend sessions. Regarding location, 40 participants (41.7%) stated that they would want to receive SMS in a clinic environment, 12 (12.5%) reported that they would want to receive support in their home, and 44 (45.8%) stated no preference. Of the 97 participants, 48.5% (47) reported that they would be open to a group or individual delivery format, or a mix of the two. About a quarter (26, 26.8%) stated a preference for an individual format, and 24 (24.7%) expressed that they would prefer a group format. About half of participants (48, 49.5%) would like to bring a family member or friend to sessions of SMS, while only 11 (11.3%) reported that they would not want to, and a further 38 (39.2%) said that they would not mind. Participants were asked to indicate whether they would be willing to receive SMS from a range of different experts/professionals. Most participants reported willingness to receive support from a nephrologist or a self-management expert external to their healthcare (68, 70.1 and 69, 71.1%, respectively; see Fig. 3).

Additional suggestions regarding how SMS should be delivered was provided by 22 participants (22.7%). Only 10.3% (10) identified online and smartphone tools (e.g., webinars, provision of materials online, online tutorials, smartphone applications) as potentially helpful, and three desired hardcopy written materials. Two participants identified difficulties inherent in rural living, with one requesting that sessions be run outside of big cities and another reporting that webinars would be good for those living in isolated areas. Two participants mentioned the importance of open communication, and of the educator being a good communicator. The importance of tailoring SMS to individuals’ needs and wants was noted by two participants. Other suggestions included: showing videos of how the kidneys work; delivering sessions via telephone; and running a program through a local hospital. The importance of delivering SMS early in the disease process was also identified.

## Discussion

The findings of this study indicate that people with CKD view self-management as complex and multi-faceted, and desire support across a range of areas. While HCPs traditionally focus on delivering CKD-related information and facts to patients [41], participants in the current study actually rated receiving support to keep a positive attitude and take care of their physical and mental health as more important. Furthermore, managing medications and learning to actively participate in healthcare (patient engagement) were rated as just as important as gaining CKD-specific knowledge. These aspects should be focussed on during specific SMS sessions, and also kept in mind during HCP-patient interactions. The 10 areas discussed in this and previous [2] work may be used as a SMS checklist when working with patients, i.e., both to establish individual patients’ SMS priorities, and to ensure that all relevant areas are addressed. The desire for additional support with many facets of self-management indicates that SMS needs are currently not being met.

Consistent with previous research, which has identified that some people with CKD are passive “receivers” of information (i.e., accept information given to them by their nephrologist), while some are active “engagers” (i.e., are aware of their needs for support and actively seek it [42]), some groups of participants in the current study expressed a stronger overall desire for SMS. Further research is required to investigate reasons for this. It is possible that those who are reporting less desire for support are already successfully self-managing, and this could be investigated using physician reports and laboratory results over time as proxies for effective self-management, as well as tools which directly assess engagement in self-management behaviour (e.g., the Chronic Kidney Disease Self-Management Instrument [43]. It is also possible (and perhaps more likely) that those who have a weaker desire for additional support may not be as actively engaged in their healthcare, and may require different and/or more intensive support to encourage them to effectively self-manage.

Past research has demonstrated that a number of the sub-groups found to have less desire for support in this study (older participants, male participants, and lower socioeconomic status (SES) participants) are less actively involved in their healthcare than their younger, female, higher SES counterparts [44, 45]. Importantly, it seems that those with lower SES, who may already be at higher risk for negative outcomes [46], may be less engaged with their healthcare, and may require more focussed efforts at PE. Furthermore, several participants conversationally expressed hopelessness with regard to whether there is anything that they can do to improve their health outcomes. This further highlights the need for increased support for people with CKD, including education about the ways in which effective self-management *can* improve health outcomes. Future research could also investigate whether these feelings are associated with decreased interest in learning more about self-management (due to feeling that it is pointless). It should also be noted that many people with CKD have multiple chronic health conditions (e.g.,[47]), and having to self-manage more than one disease further increases complexity.

Previous research has indicated that people with later stage CKD are eager to learn and wish they had understood the importance of self-management earlier [38], and in the current study those who had CKD longterm (10+ years) expressed a stronger overall desire for support than those who have been living with CKD for a shorter period. Although intensive education is often delivered to CKD patients later in the disease process, typically before initiation of RRT (e.g., information about treatment options, [48]), effective self-management during the earlier stages of CKD is crucial [49] and, as such, it is important that research is conducted regarding ways in which those who are in earlier stages of the disease can be motivated to effectively manage their illness. While self-management of CKD in stage 5 (end-stage) requires adhering to more and stricter treatment regimens (e.g., dietary and fluid restrictions, medications, RRT), the items included in the current study were carefully chosen so as to apply to patients at all stages of the CKD process. CKD stage was not directly assessed in this self-report study (with “time since diagnosis” collected instead), as people with CKD are often unaware of the stage of their CKD, or even that they have CKD at all [50, 51]. Patient engagement has been explored in chronic disease self-management, and a recent review of the literature revealed that interventions show promise with regard to improving patient engagement *and* patient outcomes, and concluded that patient engagement is an important predictor of outcomes and should be fostered [52]. Within the renal specialty, the multidisciplinary HCP team are well-placed to bring about the shift towards fostering patient engagement in their practice.

The preferences reported by participants in this study regarding how structured SMS should be organised can help to guide delivery logistics. Participants reported flexibility regarding location and format, but timing of sessions may prove more problematic due to differences in availability between those who are working and not working. This highlights the need to ensure that SMS is being offered at times that the individual patients being targeted are actually available to attend. Participants identified the difficulties inherent in living rurally, but also the opportunities that technology provide in terms of using tools such as the internet and telephone sessions to get around both of these challenges. The willingness of this sample to use such tools is encouraging, as it shows that a shift may be beginning to occur in this patient group, unlike in the past when chronic disease populations have used the internet less than healthy adults [53] and did not wish to use tools like mobile phones [54] or the internet to receive support with their disease [55, 56]. It is worth noting, however, that participants who identified the internet as a useful tool through which to receive SMS in this study were respondents to the online version of the survey, who may be more engaged with modern technology.

A variety of modes of information-delivery were identified as helpful (written materials, online contact, videos, etc.), which is consistent with previous findings that people with chronic disease appreciate multi-modal learning activities [57]. Participants noted the need for SMS to be tailored to individual wants and needs, rather than a “one-size fits all” approach. Interestingly, most participants reported that they would like to receive SMS from a nephrologist or a self-management expert external to their healthcare team. Only just over half of participants indicated willingness to receive this support from a nurse, which may indicate a lack of understanding of the role that nurses have within the multidisciplinary team.

The current study was limited with regard to sample size and sampling frames. However, this exploratory study was among the first to investigate the desires of patients with CKD for receiving SMS, including the manner in which they would like to receive this support, using a measure based on current literature [2]. As no standardised, validated assessment measure currently exists to assess desire for SMS in CKD, the researchers developed one which demonstrated high face and content/construct (as judged by academic and clinical experts in the field) validity and used language at an eighth grade reading level.

## Conclusions

People with CKD require additional support across the self-management spectrum. The findings from this study highlight the need for person-centred care and patient engagement in the renal world, as different groups of patients vary in their overall enthusiasm for learning more about effective self-management. Prior to this study, there was very little literature regarding *how* people with CKD would like to receive SMS [2]. The findings reported here not only provide support for including a wide range of aspects of self-management in self-management programs (rather than focussing solely on imparting CKD-specific information), but may also be used to guide the implementation of self-management programs with regard to format, location, mode, and educator. The results of this study indicate that a multi-modal, highly individualised (person-centred) framework for supporting self-management in CKD is required.

## Additional files

Additional file 1: Survey instrument. (PDF 152 kb)

## Abbreviations

CKD: Chronic kidney disease
SMS: Self-management support.
