# Supplementary material for: Person-centred care in chronic kidney disease: a cross-sectional study of patients’ desires for self-management support
Source: BMC Nephrol. 2017 Jan 13;18:17. doi: 10.1186/s12882-016-0416-2 (PMC5237219; doi:10.1186/s12882-016-0416-2)
Supplement: Additional file 1: — Survey instrument. (PDF 152 kb) [file 12882_2016_416_MOESM1_ESM.pdf]

Participant Code: P1: P

Demographic Information

	Please tick (✔)
**Gender**	☐ Male[1]<br>☐ Female[2]
**Age**	________________years
**Years of education**	________________years
**Highest educational qualification obtained**	☐ No Formal Education[0]
	☐ Less than Grade 10 Equivalent[1]
	☐ Grade 10 or Equivalent[2]
	☐ Grade 12 or Equivalent[3]
	☐ TAFE Qualification/Certificate/Diploma[4]
	☐ Undergraduate Degree (Bachelors)[5]
	☐ Masters Degree[6]
	☐ Doctoral Degree (including PhD)[7]
**Employment Status**	☐ Unemployed[0]
	☐ Casual Work[1]
	☐ Part Time Work[2]
	☐ Full Time Work[3]
	☐ Retired[4]
	☐ Other (Describe)[5]:
**Occupation (only complete if currently employed)**	Describe:
**Annual Household Income**	☐ Less than $20,000[0]
	☐ $20,000 – $39,99[1]
	☐ $40,000 – $59,999[2]
	☐ $60,000 – $79,999[3]
	☐ $80,000 – $99,999[4]
	☐ $100,000 – $119,999[5]
	☐ $120,000 +[6]
	☐ Don't know / Would rather not say[7]
**Approximately how long ago were you diagnosed with kidney problems?**	_____________years ____________months
	☐ Don't know

Participant Code: P1: P

The following section asks about the sort of self-management support you would like for your chronic kidney disease (CKD).

Please rate from ***0 = Not at all*** to ***10 = Very much*** how much you would like to learn about the following areas of self-management of your CKD. Please place a tick (✔) in the box that corresponds with your answer (see example, below).

EXAMPLE:

**0** *Not at all*	**1**	**2**	**3**	**4**	**5** *Don't mind*	**6**	**7**	**8**	**9**	**10** *Very much*
				✔						

---

1. **CKD-specific knowledge** (e.g., information about what the kidneys do, how they function, what happens in CKD, treatment options, ways to delay dialysis, etc.):

**0** *Not at all*	**1**	**2**	**3**	**4**	**5** *Don't mind*	**6**	**7**	**8**	**9**	**10** *Very much*

---

2. **Setting a routine and planning ahead**
(e.g., using reminder systems, getting into self-management habits):

**0** *Not at all*	**1**	**2**	**3**	**4**	**5** *Don't mind*	**6**	**7**	**8**	**9**	**10** *Very much*

---

3. **Actively participating in healthcare** (e.g., learning to change self-management based upon results, working with doctors regarding disease and treatment):

**0** *Not at all*	**1**	**2**	**3**	**4**	**5** *Don't mind*	**6**	**7**	**8**	**9**	**10** *Very much*

---

4. **Keeping a positive attitude and taking care of mental and general physical health**
(e.g., avoiding anxiety and depression, staying positive, staying physically healthy):

**0** *Not at all*	**1**	**2**	**3**	**4**	**5** *Don't mind*	**6**	**7**	**8**	**9**	**10** *Very much*

---

5. **Changing lifestyle** (e.g., sticking to your fluid and dietary guidelines, exercising):

**0** ***Not at all***	**1**	**2**	**3**	**4**	**5** ***Don't mind***	**6**	**7**	**8**	**9**	**10** ***Very much***

----

6. **Managing medications** (e.g., understanding why they're prescribed, side effects you might have, what might happen if you do not take them, taking them as prescribed):

**0** ***Not at all***	**1**	**2**	**3**	**4**	**5** ***Don't mind***	**6**	**7**	**8**	**9**	**10** ***Very much***

----

7. **Noticing and treating signs related to CKD** (e.g., noticing symptoms of CKD and knowing how to treat them, knowing what causes symptoms):

**0** ***Not at all***	**1**	**2**	**3**	**4**	**5** ***Don't mind***	**6**	**7**	**8**	**9**	**10** ***Very much***

----

8. **Keeping social and occupational roles**
(e.g., continuing to work, continuing hobbies, sustaining relationships and home roles):

**0** ***Not at all***	**1**	**2**	**3**	**4**	**5** ***Don't mind***	**6**	**7**	**8**	**9**	**10** ***Very much***

----

9. **Getting social support** (e.g., support from family and friends, getting involved with community groups such as support groups):

**0** ***Not at all***	**1**	**2**	**3**	**4**	**5** ***Don't mind***	**6**	**7**	**8**	**9**	**10** ***Very much***

----

10. **How to build good relationships with your health care providers**
(e.g., doctors, nurses, clinic staff, allied health professionals):

**0** ***Not at all***	**1**	**2**	**3**	**4**	**5** ***Don't mind***	**6**	**7**	**8**	**9**	**10** ***Very much***

----

11. **Are there any other areas of self-management that you would like to learn more about? (Describe)**

____

____

____

____

____

Please label the topics below from 1 to 11 to indicate how much you would like to learn about each option.

1** = the topic your would like to learn about the **MOST

11** = the topic you would like to learn about the **LEAST

TOPIC	RATING
Noticing and treating signs related to CKD	
Changing your lifestyle to help your CKD	
Keeping your social and occupational roles, despite your CKD	
Building good relationships with your healthcare providers (e.g., doctors, nurses)	
Learning more about your kidneys and about CKD	
Being an active participant in your healthcare	
Getting support from friends and family	
Getting into good routines to help manage your CKD	
Keeping a positive attitude and looking after your mental and physical health	
How to manage medications	
Other (Describe: ____________________ ____________________)	

Thank you!
We have just a few more questions for you about your preferences for receiving self-management support.

1. When (in the week) would you be able to attend sessions of a self-management program? (Tick as many as apply)
   - ☐ During the week during the day (between the hours of 9am and 5pm)[1]
   - ☐ During the week in the evening (after 5pm)[2]
   - ☐ On weekends[3]

2. Where would you be willing to attend sessions of a self-management program? (Tick as many as apply)
   - ☐ Outside your home (e.g., at the clinic)[1]
   - ☐ At home[2]

3. How would you like the face-to-face self-management program to be run? (Tick one option)
   - ☐ Individually (one-on-one with expert)[1]
   - ☐ Group (with others with CKD + expert)[2]
   - ☐ No preference[3]

4. Would you like for a friend or family member to attend the program with you? (Tick one option)
   - ☐ Yes[1]
   - ☐ No[2]
   - ☐ Wouldn't mind[3]

5. Who would you like to help you learn about self-management? (Tick as many as apply)
   - ☐ Your normal doctor (GP)[1]
   - ☐ A nephrologist (kidney specialist doctor)[2]
   - ☐ A nurse[3]
   - ☐ An expert in CKD self-management[4]
   - ☐ Other[5] (detail: ___________________________)
   - ☐ No preference[6]

6. **Do you have any other suggestions for *how* you think a self-management program for CKD should be run? (Please describe)**

____

____

____

____

____

Thank you! Your participation is greatly appreciated. ☺
